# Kraurosis Vulvæ

**Published:** 1890-10

**Authors:** Charles N. Smith

**Affiliations:** Toledo, Ohio


### KRAUROSIS VULVÆ.

By CHARLES N. SMITH, M. D., Toledo, Ohio.

UNDER the name of "Serpiginous Vascular Degeneration," Mr. Lawson Tait,[1] in 1877, described a diseased condition of the nymphæ, which was essentially a progressive atrophy of these parts, attended with the appearance of exquisitely sensitive areas of a brick-red to purplish color upon mucous membrane. These sensitive areas had a tendency to disappear after a time and appear in another situation, or extend in a serpiginous manner. This extension generally continued until the whole inner surface of the labia minora and the vestibule had been traveled over. These spots were so sensitive to pressure that intercourse could not, as a general thing, be tolerated. The disease was one of long duration ; little amenable to treatment ; recovery generally spontaneous ; and resulting in atrophy of the nymphæ and narrowing of the vaginal opening.

This condition generally occurred, as observed by Mr. Tait, at or after the climacteric, although he reports one case in a girl of seventeen years.

In his recent work, Mr. Tait says[2] :

> The nymphæ are also subject to a peculiar and atrophic change, which occurs generally at or after the climacteric period. It is a very distressing complaint, and one of the most intractable with which we ever have to deal. It is very often, but by no means always, associated with vascular caruncle of the urethra. . . . This affection has been alluded to by Simpson and various other authors, but no description which I have seen includes all the facts that may be observed in connection with it. It is always confined, in my experience, to the mucous membrane on the inner surface of the nymphæ, and is never met with on the labia majora or in the vagina higher than the vestibule.

From other cases, which I shall refer to later in this article, it will be seen that the disease does extend to the labia majora, although not observed there by Mr. Tait.

As to the course and final termination of this disease, Mr. Tait further says[3] :

> It is, in fact, a progressive atrophy of the mucous membrane, the last textures affected being the blood vessels and nerves ; for when the process has been completed, the pain ceases, the redness disappears, and

1. Diseases of Women, 1877, page 43.
2. Diseases of Women and Abdominal Surgery, 1889, page 51, *et seq.*
3. Op. cit., page 53.

nothing remains but a vestibulum vagina, so narrow that incredulity may be excused when the patient states that she has borne children.

The treatment recommended by Mr. Tait is the application of strong carbolic acid to the red spots, and the insertion between the labia, at bed-time, of a tampon wet with a saturated solution of neutral acetate of lead in glycerine.

Edis[1] describes this disease, but throws no new light upon it. In fact, his article is merely a description of the disease as observed by Tait. Matthew D. Mann describes the disease as seen by Tait, and says[2]:

I have never met with any cases of this disease, and am unacquainted with any reference to it in American medical literature.

Skene has undoubtedly met with the disease in its last or atrophic form, as in speaking of pruritus vulva he says[3]:

In the majority of cases of this kind that have come under my observation, the skin has been bleached, in spots appearing whiter than the normal skin. It has also the normal elasticity. To the touch it seems harder and less flexible, but what these changes are, and whether they are related to the pruritus, are questions which have not been yet answered.

In 1885, Breisky,[4] of Prague, published an account of twelve cases of this peculiar disease which had come under his observation. He gave it the name of "Kraurosis Vulvæ," or shrinking of the vulva. Most of the cases observed by Breisky were evidently in the last stage of the disease, as he makes no mention of the presence of the red spots except in one case.

In a recent paper,[5] Dr. H. H. Ohmann-Dumesnil reports thirty-five cases which he has collected from various sources, including three cases observed by himself. Breisky's cases are included, but no reference is made to those seen by Tait. Robert F. Weir first described a case of this disease in March, 1885.[6] In these thirty-five cases the presence of thickened areas or plaques of various colors, red, gray, milky, pearly or opaline, is mentioned as occurring in nearly all. Atrophy of some part or parts of the vulva was present in all. In some of them the labia majora were affected, which is contrary to the observations of Tait. Pruritus, a condition not mentioned by Tait, was present in fifteen, and ranged in

1. Diseases of Women, 1881, page 390, et seq.
2. American System of Gynecology, Vol. I., page 509, et seq.
3. Diseases of Women, 1889, page 95, et seq.
4. *Centralblatt für Gynäkologie*, June 6, 1885.
5. New Orleans *Medical and Surgical Journal*, March, 1890.
6. *New York Medical Journal*, March, 1875.

severity from moderate to intense. Of the thirty-five cases, four had suffered from syphilis. Gonorrhea had occurred in two. Leucorrhea, a condition present in all of Tait's cases, is mentioned as being present in eleven cases.

Treatment was generally successful in those cases which were seen in the earlier stages, but was unsuccessful in the atrophic stage. The treatment consisted in curetting the spots, and in the application of liquor ferri sesquichloridi, lotions of salicylic acid, pyrogallic acid and acetic acid.

As to the frequency of the disease, Ohmann-Dumesnil says:

The fact that there have been but few cases of this character reported, does not argue that it is a rare condition by any means. In fact, I am inclined to the belief that it is rather frequent, and that it is simply owing to inattention that more of a similar character have not been reported. It is only lately that any considerable degree of attention has been drawn to this disease in medical journals, and all the literature given so far has consisted of short abstracts of the articles quoted in this paper, and of Dr. Heitzmann's cases. When the condition becomes better known, the number of cases will, no doubt, multiply, and what is now looked upon as an unusual disease, will be frequently seen and noted.

I now add one more case to those already reported:

Mrs. A., aged fifty-two, married, and the mother of two children, the younger aged sixteen, consulted me in April, 1888. She was of a marked nervous temperament, slight and delicate figure, but apparently in good health. She had never had syphilis nor gonorrhea. Six years before, and four years prior to the cessation of menstruation, she first noticed a pruritus vulva, attended with pain and scalding upon urination. The pruritus gradually increased for about one year, when she consulted a physician, who found and removed a urethral caruncle. Following the operation there was relief from the pain and scalding upon urination, but no abatement of the pruritus. On consulting a second physician, several months later, she was told that her trouble was undoubtedly the result of diabetes. She was therapeutically and dietetically treated for diabetes for nearly a year, and was given carbolized washes and various ointments for the vulva. The carbolized washes somewhat relieved the pruritus, although it was always present in some degree. This physician made no local examination and never examined the urine for sugar, although he was positive in his diagnosis of diabetes,

I carefully examined several specimens of her urine, obtained at different times, and found no trace of sugar.

On examination of the vulva a most peculiar condition was found. The hairs on the parts were few, harsh and gray. The vulva was slightly gaping. The labia majora were shrunken, inelastic and composed almost entirely of integument, the subcutaneous tissue having nearly disappeared. A little over one-half of the lower or posterior portions of the labia minora were atrophied, while the upper portions were natural in size and appearance, except for the purple spots mentioned below. The vaginal opening was so narrowed that only the small, virgin size of Sims' speculum could be inserted. About one-fourth of an inch below the meatus urinarius was an exquisitely sensitive patch of dark purple color, a little over one-fourth of an inch in width, and extending downward and to the right for about one-half of an inch. Another similar spot, but smaller, was found on the inner or vaginal surface of the upper portion of the left labium minus. Small and exceedingly thin membranous tags were all that remained of the hymen. The clitoris was normal. The integument of the lower portion of the labia minora and on the inner surface of the labia majora was dry, smooth, shining, and bluish-white in color, with here and there a few small thickened spots of a milky-white color. The spots, and the atrophied areas generally, were not so sensitive to pressure as were the red spots. In fact, the kraurotic changes seemed to have been nearly completed in the lower portion of the vulva, while they were still progressing in the upper portion.

The uterus measured a plus two inches in depth. A slight thin discharge escaped from the os. Thinking that the pruritus might possibly be caused, or at least aggravated, by the discharge, I inserted into the vagina, or had the patient do so, twice a day, a dry absorbent cotton tampon to collect the discharge and prevent its escape into the vulva. This was continued for two weeks with no result.

Having, by this time, decided that nearly the whole trouble arose from the two purple spots, I applied to them equal parts of tincture of iodine and carbolic acid. This treatment resulted in a considerable diminution of the pruritus, but did not remove the spots. I then applied pure carbolic acid, which caused excruciating pain, but was, in a short time, followed by complete relief of the pruritus. These applications were continued from time to time, until the spots were entirely destroyed, or at least until they disappeared. Following the disappearance of the spots was a considerable, but not total, relief from the pruritus. Gradually the pruritus grew less and less until it, too, disappeared. In the two years which have elapsed, since the cessation of treatment, there has been no return of the sensitive spots or of the pruritus.

